# Practical Application of Novel Test Methods to Evaluate the Potency of Botulinum Toxin: A Comparison Analysis among Widely Used Products in Korea

**DOI:** 10.3390/toxins13120833

**Published:** 2021-11-23

**Authors:** Ji-Yeon Hong, Jong-Hee Kim, Jung-Eun Jin, Sun-Hye Shin, Kui-Young Park

**Affiliations:** 1 Department of Dermatology, Chungnam National University Sejong Hospital, Sejong 30099, Korea; itching119@gmail.com; 2 NABOTA Research and Development Team, Daewoong Pharmaceuticals, Seoul 06973, Korea; hee0902@daewoong.co.kr (J.-H.K.); jungeun@daewoong.co.kr (J.-E.J.); 3 Department of Dermatology, College of Medicine, Chung-Ang University, Seoul 06973, Korea; seekfresh@caumc.or.kr

**Keywords:** botulinum toxin, potency, LD_50_, endopeptidase, rapid detection

## Abstract

The safe and effective dosing of botulinum neurotoxins (BoNTs) requires accurate and reliable methods to measure their potency. Several novel methods have been introduced over the past decade; however, only few studies have compared the potency of BoNT products with that of the LD50 and other alternative assays. Therefore, the objective of this study was to comparatively evaluate widely used BoNT products using various test methods. Four types of BoNTs (prabotulinumtoxin A, onabotulinumtoxin A, neubotulinumtoxin A, and letibotulinumtoxin A) were used in this study. The estimated potency was assessed using the LD50 assay, and the total BoNT type A protein levels were measured using the enzyme-linked immunosorbent assay (ELISA). The in vitro efficacy of the BoNTs was determined using fluorescence resonance energy transfer (FRET) and surface plasmon resonance (SPR) assays. The results showed differences in the total amount of BoNT protein and the cleavage activity of SNAP-25 within all types of BoNTs. The SPR study seemed to be useful for evaluating the potency by specifically measuring intact 19S neurotoxin, and these results provide new insights for assessing different BoNT products.

## 1. Introduction

The therapeutic and cosmetic applications of botulinum neurotoxins (BoNTs) have progressed steadily over the last 30 years since the FDA approved its use for the treatment of strabismus in 1989. BoNTs are produced by various *Clostridium* species and were classified into different serotypes based on their immunological properties. Recently, with the development of genomic sequencing techniques, novel BoNT serotypes and BoNT-like toxins from non-clostridial species have been discovered [1,2,3,4]. BoNTs exert their neurotoxic activity by blocking neuromuscular conduction via the inhibition of presynaptic release of acetylcholine into neuromuscular junction [5]. Among the various types of BoNTs, type A and type B are most commonly indicated for muscle spasticity, excessive sweating, migraines, overactive bladder, and facial wrinkles [6]. For BoNT products utilized for medical purposes, the biological activity of the active component must be accurately determined for each batch using suitable and highly precise assay systems. Due to its high sensitivity, the in vivo mouse LD50 assay was adopted by all manufacturers to evaluate the potency of BoNT [7]. It assesses the amount of toxin needed to kill 50% of the mice when the preparation is injected intraperitoneally.

The LD_50_ assay used for testing each batch of BoNT requires at least 100 mice, provoking ethical concerns over animal use. In addition, it takes 2–4 days to elicit results, delaying the overall manufacturing process. It requires both applicable laboratory settings and trained personnel [8]. This emphasizes the constant demand and production of BoNTs, increasing pressure on the need to develop and validate quicker and easier alternative methods to the LD_50_ assay for potency and quality control tests.

Several novel methods for the detection and measurement of BoNT have been developed, including the enzyme-linked immunosorbent assay (ELISA), endopeptidase mass spectrometry, and protease activity assay [9,10,11]. At present, there are few published data which compare the amount and potency of BoNT products with the LD_50_ assay and other alternative assays. Here, we report a comparison analysis of four widely used BoNT products (prabotulinumtoxin A (Nabota^®^, Daewoong Pharmaceutical Co. Ltd., Seoul, Korea), onabotulinumtoxin A (Botox^®^, Allergan, Inc., Irvine, CA, USA), neubotulinumtoxin A (Meditoxin^®^, Medytox, Inc., Seoul, Korea), and letibotulinumtoxin A (Botulax^®^, Hugel, Inc., Seoul, Korea)) using the LD_50_ assay, ELISA, the fluorescence resonance energy transfer (FRET) assay, and the surface plasmon resonance (SPR) test.

## 2. Results

### 2.1. Estimated Potency for Each BoNT/A Product via LD_50_ Assay

The potency of each BoNT product was calculated based on the mouse LD_50_ units observed in each group, defined as the doses that cause 50% death in 10 mice within 72 h following intraperitoneal injection. Furthermore, 1 mL of serially diluted BoNT products (total concentrations of 26.86, 20.20, 15.20, 11.43, 8.60, 6.47, and 4.86 U/mL) were injected into the mice. The calculated potency results by probit analysis described in the European Pharmacopeia were as follows: prabotulinumtoxin A, 99.87%; onabotulinumtoxin A, 101.23%; neubotulinumtoxin A, 105.03%; and letibotulinumtoxin A, 118.87%.

### 2.2. Measurement of Total BoNT/A Protein Amounts by Sandwich ELISA

Sandwich ELISA was carried out to determine the total amounts of botulinum toxin proteins, either active or inactive, present in each vial of the indicated products, which is required to calculate the toxin efficacy value corresponding to the same toxin dose across the samples. Sandwich ELISA colorimetric detection methods were used to objectively quantify the total amount of BoNT/A proteins present in each product vial. Considering batch variations, the BoNT/A samples of 5–6 different batches were included in this study.

**Figure 1:**
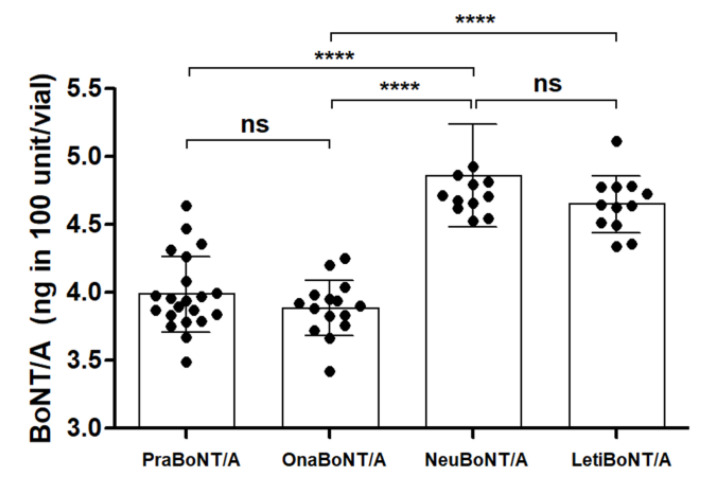
Graph summarizing the total amounts of BoNT/A protein present in each of the 100 unit vials indicated. Each dot represents one 100 unit vial of the products. *n* = 21 for Prabotulinumtoxin A (PraBoNT/A; Batch numbers used, 991451, 991452, 991453, 995158, 995159, and 995160), *n* = 15 for Onabotulinumtoxin A (OnaBoNT/A; Batch numbers used, C3312 C2F, C3603 C2, C3718 C3, C4049 C3, and C3718 C3F), *n* = 13 for Neubotulinumtoxin A (NeuBoNT/A; Batch numbers used, FAA1559, FAA15110, FAA16081, FAA16107, FAA16108, and FAA16090), and *n* = 13 for Letibotulinumtoxin A (LetiBoNT/A; HUA15030, HUA15058, HUA15162, HUA15195, HGA17021, and HGA17003). The bars represent average ± standard deviation (SD). **** *p*-value < 0.0001; ns = non-significant.

The overall amount of BoNT/A protein within a 100-unit vial of each product was detected via the sandwich ELISA (Figure 1). The total amount of BoNT/A protein contained in prabotulinumtoxin A (3.98 ± 0.28 ng) was similar to that of onabotulinumtoxin A (3.88 ± 0.20 ng), and the total amount of BoNT/A protein in neubotulinumtoxin A (4.86 ± 0.37 ng) was similar to that of letibotulinumtoxin A (4.64 ± 0.21 ng). The difference in the protein amounts between pra/onabotulinumtoxin A and neu/letibotulinumtoxin A was statistically significant (*p* < 0.0001).

### 2.3. FRET-Based Potency Assay

**Figure 2:**
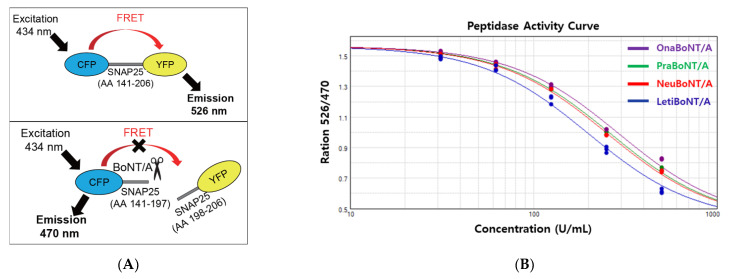
(**A**) Image depicting the principle of FRET-based analysis used in this study. (**B**) FRET-based BoNT/A activity assay. The *y*-axis value, OD 526 nm (reflecting the uncleaved SNAP- 25)/OD 470 nm (reflecting the cleaved SNAP-25), is inversely correlated to toxin activity. Prabotulinumtoxin A (PraBoNT/A), green; Onabotulinumtoxin A (OnaBoNT/A), purple; Neubotulinumtoxin A (NeuBoNT/A), red; Letibotulinumtoxin A (LetiBoNT/A), blue; Standard (the mean of OnaBoNT/A). *n* = 12 for Prabotulinumtoxin A, *n* = 9 for Onabotulinumtoxin A, Neubotulinumtoxin A, and Letibotulinumtoxin A.

We used a commercially available FRET-based BoNT/A activity measurement kit (BoTest^®^). As depicted in Figure 2A, to exploit FRET, the internal fragment of SNAP-25, which is recognized and cleaved by BoNT/A, was conjugated to two fluorescent proteins in this assay, cyan fluorescent protein (CFP) and yellow fluorescent protein (YFP) [12]. Thus, cleavage of the CFP-SNAP-25 AA141-206-YFP protein by active BoNT/A results in a CFP signal (Figure 2A, upper panel). However, no cleavage of the CFP-SNAP-25 AA141-206-YFP protein led to the YFP signal (Figure 2A, lower panel).

Using this assay, we quantified the peptidase activity of BoNT/A within each vial of the products. Five different doses of each replicate (0, 62.5, 125,250, and 500 units) were included to obtain reliable quantification data reflecting the toxin activity. The activity curves for prabotulinumtoxin A, onabotulinumtoxin A, neubotulinumtoxin A, and letibotulinumtoxin A were generated using the ratio of OD 526 nm reflecting the uncleaved SNAP-25 (FRET thus the YFP signal) to OD 470 nm reflecting the cleaved SNAP-25 (no FRET, thus the CFP signal) (Figure 2B), which was used to calculate the toxin efficacy using the 4PL regression model (Table 1, FRET data).

### 2.4. SPR-Based Potency Assay

**Figure 3:**
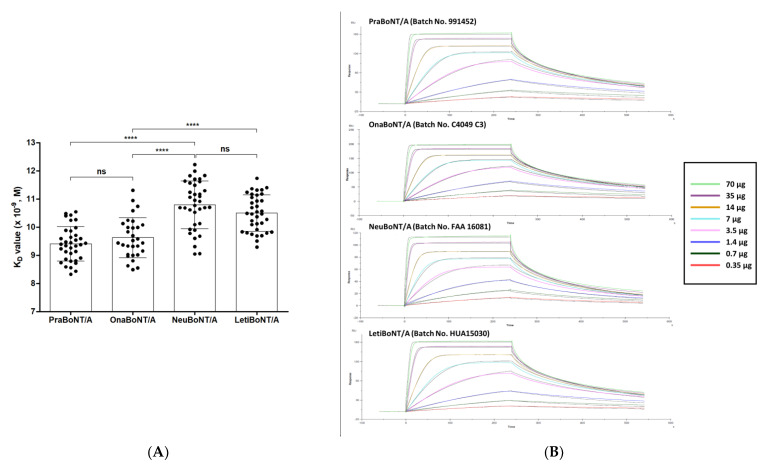
SPR-based BoNT/A efficacy measurements. (**A**) Results of the kinetic assay. Graph summarizing K_D_ values reflecting the toxin activity. Each dot represents a 100-unit vial of the products. *n* = 36 for prabotulinumtoxin A (PraBoNT/A; Batch numbers used, 991451, 991452, 991453, 995158, 995159, and 995160), *n* = 30 for onabotulinumtoxin A (OnaBoNT/A; Batch numbers used, C3312 C2F, C3603 C2, C3718 C3, C4049 C3, and C3718 C3F), *n* = 36 for neubotulinumtoxin A (NeuBoNT/A; Batch numbers used, FAA1559, FAA15110, FAA16081, FAA16107, FAA16108, and FAA16090), *n* = 36 for Letibotulinumtoxin A (LetiBoNT/A; Batch numbers used, HUA15030, HUA15058, HUA15162, HUA15195, HGA17021, and HGA17003). The bars represent average ± standard deviation (SD). ns, not significant; **** *p* < 0.0001. Unpaired two-tailed *t*-tests. (**B**) Representative SPR sensograms from the anti-SNAP-25 mAb binding to the SNAP-25 AA 1–197 fragment (0.35–70 μg/mL) over a range of high (green) and low (red) concentrations: 0.35, 0.7, 1.4, 3.5, 7, 14, 35, and 70 μg/mL were tested. The sensograms were normalized to fit a 1:1 binding model using the Biacore T200 evaluation software (GE Healthcare Life Sciences). Prabotulinumtoxin A, PraBoNT/A; onabotulinumtoxin A, OnaBoNT/A; neubotulinumtoxin A, neuBoNT/A; and letibotulinumtoxin A, LetiBoNT/A.

We conducted SPR-based in vitro potency analysis with the four BoNT/A products and compared them with the data obtained from FRET-based efficacy testing. Similar to the FRET-based assay, SNAP-25, the substrate of BoNT/A, was used to quantify toxin activity [3]. Simply put, the full-length SNAP-25 protein was immobilized on the SPR sensor chip, where the botulinum toxin products were run over the SNAP-25 protein coated on the sensor chip for the recognition and cleavage of SNAP-25. We used a monoclonal antibody (mAb) that specifically recognizes amino acids 183–197 of SNAP-25 because the active botulinum toxin cleaves the full-length SNAP-25 to SNAP-25 AA 1–197 and SNAP-25 AA 198–206. This epitope sequence is part of the N-terminal cleaved fragment of SNAP-25. A titrated series of mAb concentrations (0.35–70 μg/mL) was run over the sensor chip to determine the apparent equilibrium dissociation constant K_D_ value that reflects the toxin activity. Representative sensograms and graphs summarizing all measurements are shown in Figure 3. The K_D_ value was calculated by determining the ratio of dissociation rate constant K_D_ to the association rate constant K_a_. In this SPR assay, the K_a_ value indicates the binding association rate between the cleaved SNAP-25 fragment AA 1–197 and the anti-SNAP-25 AA 183–197 mAb as well as the K_D_ value for their binding dissociation rate (Table 1, K_D_ data).

The K_D_ values obtained, which reflect the toxin activity for each product, are as follows: 9.40 ± 0.61 (prabotulinumtoxin A), 9.62 ± 0.71 (×10^−9^ M, onabotulinumtoxin A), 10.79 ± 0.85 (neubotulinuntoxin A), and 10.50 ± 0.64 (letibotulinumtoxin A). Similar to the ELISA results, the difference in K_D_ values between pra/onabotulinumtoxin A and neu/letibotulinumtoxin A was statistically significant (*p* < 0.0001), while the K_D_ values of prabotulinumtoxin and onabotulinumtoxin were similar.

## 3. Discussion

The constant demand and resultant hike production of BoNT products suggest replacing the mouse LD50 assay due to the ethical issues over animal use and scientific concerns over the precision of the method. Therefore, a number of alternative in vivo, ex vivo, and in vitro analytical methods have been developed and tested for validation. However, few studies have compared each BoNT product with LD50 assay and other alternative methods of individual merits and demerits [13,14]. In this study, we reported the amount of BoNT protein and cleavage activity of SNAP-25 within widely used BoNT/A products.

In the ELISA study, we determined the respective amounts of overall neurotoxin per 100 unit. The results showed that neubotulinumtoxin A and letibotulinumtoxin A contained relatively larger amounts of neurotoxins than the standard onabotulinumtoxin A, making onabotulinumtoxin the most prevalent specific biological activity (unit/ng neurotoxin), followed by prabotulinumtoxin. It is hypothesized that some parts of neurotoxins in neubotulinumtoxin and letibotulinum may be inactive during freeze-drying or during reconstitution with saline.

Knowing both the amount of total neurotoxin and active neurotoxin in these preparations is important because of two reasons. First, the accompanying impurities within the products may stimulate the immune reaction against the active therapeutic agent [15]. Like other biologic drugs, the development of immunogenicity after long-term treatment with BoNTs is one of the major clinical concerns. Recently, Samadzdeh et al. reported that the higher protein load of BoNTs is associated with a higher incidence and prevalence of neutralizing antibody formation and that the clinical response is also dependent on the protein load of the preparation [16]. Second, the total injected amount of BoNT along with denatured or inactive neurotoxin determines the intensity and possibility of an immune response [17]. Notably, the presence of residual inactive neurotoxin in neubotulinumtoxin and letibotulinumtoxin can be associated with the stimulation of neurotoxin neutralizing antibodies in some patients due to the higher dose of injected antigen. To define the link between the excess protein and potential immunogenicity, blood tests for neutralizing antibody detection may be necessary in the future study.

Despite the differences in the units per mass of neurotoxin protein defined in ELISA studies, relevant studies have already revealed that the clinical efficacy of onabotulinumtoxin and neubotulinumtoxin are comparable [14,18]. When comprehensively analyzing the overall results, neubotulinumtoxin showed a similar in vivo potency in the LD50 assay with onabotulinumtoxin, assuming that neubotulinumtoxin’s larger amount of neurotoxin per unit compensated for its potential denaturation. Together, these results demonstrate that the amount of neurotoxin in neubotulinumtoxin and onabotulinumtoxin exhibited similar activity in the clinical environment to that in the mouse LD_50_ assay.

In contrast, letibotulinumtoxin showed significantly greater potency per unit than standard onabotulinumtoxin in both in vivo and in vitro studies. Based on these data, we can assume that injecting the same dose of letibotulinumtoxin may result in a relatively stronger therapeutic effect compared to the other products. A more potent paralytic effect requires the skills of experienced practitioners to avoid unpleasant results of overcorrection, especially in cosmetic fields. Delicate dose adjustment and optimal dilution should be determined according to the treatment site and the expected outcome.

Prabotulinumtoxin showed a relatively low K_D_ value similar to that of onabotulinumtoxin, which correlates with the higher potency in SPR studies. Unlike other studies, such as Western blotting, which inevitably detect the denatured parts of neurotoxins, the SPR assay selectively determines the potency of intact 19S neurotoxin with its higher sensitivity [19,20]. BoNTs can affect the target cells via disassociation of progenitor toxins complex including 19S neurotoxin and release of free 150 kDa neurotoxin [21]. Therefore, the differences in potency of BoNT products depend of the amount of active toxin proteins. From the SPR assay, we estimated that prabotulinumtoxin and onabotulinumtoxin contain lower amounts of denatured/inactive proteins. Simply put, the antigen dose, which is responsible for the formation of neutralizing antibodies that may result in treatment failure, is lower [22,23].

Since the therapeutic effects of BoNT/A are not permanent, periodic injections are necessary to maintain these benefits [24]. For patients who receive frequent dosing over a long period, immunogenicity may be a concern [25]. Although previous studies have confirmed that prabotulinumtoxin A, onabotulinumtoxin A, neubotulinumtoxin A, and letibotulinumtoxin A were comparable in terms of clinical efficacy [26,27,28], differences in their specific biological potency, complexing proteins, and inactive toxin contact can be related to their immunogenicity, therapeutic outcome, and side effect profile. Therefore, physicians should be aware of relevant data which reveal how each product differs in order to ensure safe and effective use.

## 4. Conclusions

Here, we report the comparative analysis of four BoNT products widely used in Asia using LD50 assay, ELISA, FRET assay, and SPR analysis. The results demonstrated differences in the total amount of BoNT protein and the cleavage activity of SNAP-25 within the four BoNT products. These preclinical results will lead to new insights into evaluating various BoNT products, enabling clinicians to perform safer and more effective procedures.

## 5. Materials and Methods

### 5.1. Toxin Preparations

The BoNT/A preparations used in our study were prabotulinumtoxin A (Batch No. 991451, 991452, 991453, 995158, 995159, 995160; Nabota^®^, Daewoong Pharmaceutical Co. Ltd., Seoul, Korea), onabotulinumtoxin A (Batch No. C3718 C3, C3718 C3F, C4049 C3, C3312 C2F, C3603 C2; Botox^®^, Allergan, Inc., Irvine, CA, USA), neubotulinumtoxin A (Batch No. FAA1505, FAA1514, FAA1552, FAA 1559, Faa15110, FAA16081, FAA16107, FAA16108, FAA16090; Meditoxin^®^, Medytox, Inc., Seoul, Korea), and letibotulinumtoxin A (Batch No. HUA15030, HUA15058, HUA15162, HUA 15195, HGA17021, HGA17003; Botulax^®^, Hugel, Inc., Seoul, Korea). Their characteristics are summarized in Table 2. The stated potencies of all BoNT/A products were 100 units, which were measured by mouse IP LD_50_ assay in accordance with the protocol by Korean Ministry of Food and Drug Safety and European Pharmacopeia.

### 5.2. LD_50_ Assay

ICR/CD-1 mice (4-week-old, female, 18–22 g each; KOATECH, Gyeonggi-do, Korea) were used for the test. All the procedures involving animals were conducted in accordance with the guidelines provided by the Institutional Animal Care and Use Committee of Daewoong Pharmaceutical Biocenter, Republic of Korea (number: IACUC-18–002). The LD50 was determined using a seven-serial dose with a dilution interval of 1.25 (total concentrations of 26.86, 20.20, 15.20, 11.43, 8.60, 6.47, and 4.86 U/mL, respectively), and 10 mice were designated per dose [29]. The mice were observed for 72 h after intraperitoneal administration of BoNT/A preparations with injection volume of 0.1 mL. The potency results were calculated by probit analysis based on the European Pharmacopeia. The estimated potency should not be less than 80% and not more than 120% of the stated potency, as prescribed by the European Pharmacopeia.

### 5.3. Sandwich ELISA

The capture antibody, horse antiserum to BoNT/A, named Botulinum Antitoxin Equine Type A (2000 IU/ampoule, National Institute for Biological Standards and Control NIBSC, Product No. 59/021), was diluted with the ELISA plate coating buffer (0.1 M sodium bicarbonate, pH 9.6) to a final concentration of 10 mIU/mL. One hundred microliters were added to each 96-well plate and incubated at 4 °C overnight. After the plate was emptied, 200 µL of the blocking solution (PBS/2% BSA) was added to each well and incubated at 37 °C for 90 min. The plate was then emptied and washed with a solution (PBS/0.05% polysorbate 20). Each vial of prabotulinumtoxin A (100 units), onabotulinumtoxin A (100 units), neubotulinumtoxin A (100 units), and letibotulinumtoxin A (100 units) were prepared by adding 1 mL of sodium chloride injection per vial. Then, 100 µL of toxin preparations was added in each well and was incubated at 37 °C for 90 min. After incubation, the plate was emptied, washed with the wash solution, and 100 µL of the primary antibody, rabbit antiserum (Meridian Life Science, Inc.) to BoNT/A (1:10,000 dilution in PBS/0.05% polysorbate 20), was added and incubated at 37 °C for 90 min. After washing with the wash solution, 100 µL of the secondary antibody, goat antiserum to rabbit immunoglobulin conjugated to horseradish peroxidase HRP (1:5000 dilution in PBS/0.05% polysorbate 20), was added to each well and incubated at 37 °C for 90 min. After washing, 100 µL of 3,3′,5,5′-tetramethylbenzidine TMB substrate solution was added to each well. The color was developed for approximately 30 min while being protected from light, and 50 µL of sulfuric acid was added to each well to stop the reaction. The absorbance measurement device SpectraMax of Molecular Devices was used at 450–540 nm, and the results were analyzed using SoftMax Pro. For quantitative analysis of each product, the standard curve values for Botulinum toxin type A diluted to concentrations of 10 ng/mL–0.156 ng/mL were calculated and applied. The test method used in this study was validated according to the International Conference on Harmonization guidelines.

### 5.4. FRET-Based Potency Assay

We detected BoNT type A (BoNT/A) cleavage activity of SNAP-25 using a FRET assay with a BoTest^TM^ detection kit (Biosentinel Catalog No. A1004). It contains a reporter comprising residues 141–206 of SNAP-25, the naturally occurring substrate of BoNT/A. The substrate was subsequently sandwiched between two fluorescent proteins: a cyan fluorescent protein (CFP) derivative and a yellow fluorescent protein (YFP) derivative. The CFP and YFP moieties form a donor-acceptor fluorescence resonance energy transfer (FRET) pair. Botulinum toxin products were incubated at room temperature for 18 h. After incubation, the reporter was excited at 434 nm, and the emission was measured at 470 and 526 nm on a SpectraMax plate reader. The relative efficacy of the BoNT/A products was calculated using the parameter logistic (4PL) regression model using SoftMax Pro v5.4 (Molecular Devices). The equation for this model is as follows:

y = d + (a − d)/(1 + (x/c)b)(1)

where x is the dose used in FRET assays, y the FRET signals, a denotes the minimum value obtained (at 0 dose), b the slope of the curve at point c, c the point on the S-shaped curve halfway between a and d, and d the maximum value obtained (at infinite dose).

### 5.5. SPR Binding Analysis

#### 5.5.1. Preparation of SNAP-25 Chips

The human SNAP-25 full-length protein (amino acid residues AA 1–206) was immobilized on the sensor chip CM5 for Biacore T200 (GE Healthcare Life Sciences). For immobilization, SNAP-25 was prepared at a concentration of 10 µL/mL using an acetate buffer (pH 4.0) and run on the chip using an HBS-EP running buffer. The analysis was conducted following the procedure provided by the manufacturer’s instructions. The target immobilization density was set at 400 RU, and the test was performed when the density was not less than 400 RU. The cleavage buffer containing 50 mM HEPES-NaOH (pH 7.4), 0.1% BSA, 10 mM DTT, 1% Tween 20, and 100 μM ZnCl_2_ was used for equilibration.

#### 5.5.2. Endoprotease Activity Using SPR Analysis

SPR analysis was performed using Biacore T200 (GE Healthcare) and HBS-EP buffer (running buffer). Samples of prabotulinumtoxin A (100 units), onabotulinumtoxin A (100 units), neubotulinumtoxin A (100 units), and letibotulinumtoxin A (100 units) were prepared by adding cleavage buffer (0.5 mL) and applied to the prepared sensor chip under the following conditions: contact time, 3600 s; dissociation time, 900 s; and flow rate, 1 μL/min. To specifically detect the cleaved SNAP-25 fragment containing AA 1–197, an anti-SNAP-25 antibody (ABIN236420, antibodies-online GmbH, Aachen, Germany) that recognizes AA 183–197 of SNAP-25 was diluted in the HBS-EP buffer to a concentration of 0.35–70 μg/mL and sequentially applied to the chip using the following conditions: contact time, 240 s; dissociation time, 300 s; and flow rate, 30 μL/min. Regeneration was performed between various concentrations of antibody loadings using a regeneration buffer containing glycine-HCl, pH 1.5 under the following conditions: contact time, 40 s; stabilization period, 30 s; and flow rate, 30 μL/min. The equilibrium dissociation constant K_D_ was calculated by determining the ratio of the dissociation rate constant K_D_ to the association rate constant K_a_. In this SPR assay, the K_a_ value indicates the binding association rate between the cleaved SNAP-25 fragment and the anti-SNAP-25 antibody and the K_D_ value for their binding dissociation rate. Therefore, K_D_ is indicative of the toxin activity.

### 5.6. Statistical Analysis

The concentration of BoNT/A in each product was determined using descriptive statistics. The LD_50_ analysis was performed using StatPlus 2009 Program (Release 5.9.8. AnalystSoft). One mouse LD_50_ was defined as one unit. LD_50_ was translated into estimated potency using the formula (U/vial: 100 U/LD_50_, estimated potency: U/vial/100 U). The ELISA data were analyzed using SoftMax Pro for quantitative measurement. In the FRET assay, the relative efficacy of BoNT/A products was calculated using the four-parameter logistic (4PL) regression model using SoftMax Pro v5.4 (Molecular Devices). For SPR analysis, sensorgram curves were fitted to an analytic model provided by GE Healthcare (Biacore T200).

A two-tailed, unpaired Student’s *t*-test for two-group comparisons was utilized for a head-to-head comparison of each product. The statistical significance was set at *p* < 0.05. All statistical analyses were performed using JMP 15 (statistical software, SAS Institute).

## Figures and Tables

**Table 1:** Assessment of the potency of botulinum toxin products.

Product Name	ELISA (ng/100 U)	FRET (Efficacy Relative to 100 U OnaBoNT/A)	Potency (%)	K_D_ (×10^−9^, M) *
Prabotulinumtoxin A	3.98 ± 0.28	111.0 ± 0.033	99.87 ± 5.29%	9.40 ± 0.61
Onabotulinumtoxin A	3.88 ± 0.20	100.0 (Standard)	101.23 ± 10.90%	9.62 ± 0.71
Neubotulinumtoxin A	4.86 ± 0.37	115.4 ± 0.034	105.03 ± 9.29%	10.79 ± 0.85
Letibotulinumtoxin A	4.64 ± 0.21	146.4 ± 0.043	118.87 ± 9.65%	10.50 ± 0.64

* *p*-value < 0.05.

**Table 2:** Summarized characteristics of botulinum neurotoxin type A preparations used in the study.

Product Name	Brand Name	Drying Method	Dosage (unit)	Composition	Excipients	Storage
PraBoNT/A	NABOTA^®^	Vacuum-dried	100 U	BoNT/A 900 kD complex	0.5 mg human serum albumin, 0.9 mg NaCl	2~8 °C
OnaBoNT/A	BOTOX^®^	Vacuum-dried				
NeuBoNT/A	Meditoxin^®^	Freeze-dried				
LetiBoNT/A	Botulax^®^	Freeze-dried				

## Data Availability

The data that support the findings of this study are available from the corresponding author upon reasonable request.
